# Anti-Typhoid Inoculation

**Published:** 1900-07-21

**Authors:** 


### ANTI-TYPHOID INOCULATION.

The statistics which have been officially collected in regard to the comparative incidence of typhoid fever on the inoculated and the non-inoculated during the seige of Ladysmith are of much interest. So far as an isolated series of observations can go they tend in a marked degree to show that the inoculation did have a marked effect in warding off the disease. Among the 12,234 officers and men under observation there were 1,524 cases of typhoid fever. Among the non-inoculated, who numbered 10,529, there were 1,489 cases, or one attack to every 7·07 men, while among the inoculated, who numbered 1,705, there were 35 cases, or one attack to every 48·70 men. The deaths from typhoid were 1 in every 32 among the non-inoculated, and 1 in every 213 among the inoculated. Thus it would appear from the report by Dr. A. E. Wright, Professor of Pathology in the Army Medical School, Netley, that the proportion on the one hand of attacks, and on the other of deaths from typhoid, was seven times smaller in the inoculated than in the un-inoculated, and it is to be borne in mind that "if the number (no doubt a considerable one) of men who had previously suffered from typhoid had been subtracted from the number of the uninoculated, as might quite legitimately have been done, the statistics would have borne an even more favourable aspect." If, however, we turn from the total mortality to the case mortality, the result, according to the figures given in this report, is the other way about. The proportion of deaths to cases was 1 in 4·52 among the not inoculated, and 1 in 4·40 among the inoculated. So far, then, as these statistics go it would appear that inoculation gives considerable protection from attack, but that those who, notwithstanding the protection thus afforded, become affected suffer more severely than those who are not inoculated. At this one need not feel surprised if we accept the hypothesis that inoculation is protective against the milder but not against the more virulent forms of infection. As a very curious outcome of the statistics we may mention that a much larger proportion of officers than of men suffered from typhoid fever, that a slightly larger proportion of officers died from the disease, and that the protective effect of the inoculations seems to have been very much less marked among the officers than among the men.

